# Integrating Multiple Database Resources to Elucidate the Gene Flow in Southeast Asian Pig Populations

**DOI:** 10.3390/ijms25115689

**Published:** 2024-05-23

**Authors:** Guangzhen Li, Yuqiang Liu, Xueyan Feng, Shuqi Diao, Zhanming Zhong, Bolang Li, Jinyan Teng, Wenjing Zhang, Haonan Zeng, Xiaodian Cai, Yahui Gao, Xiaohong Liu, Xiaolong Yuan, Jiaqi Li, Zhe Zhang

**Affiliations:** 1 National Engineering Research Center for Breeding Swine Industry, Guangdong Provincial Key Lab of Agro-Animal Genomics and Molecular Breeding, College of Animal Science, South China Agricultural University, Guangzhou 510642, China; guangzhenli6228@126.com (G.L.); yuqiangliu123@126.com (Y.L.); wershinecho11@163.com (X.F.); saradiao@126.com (S.D.); zhongzhanming204@163.com (Z.Z.); bekelejaiceyon@mail.ru (B.L.); kingyan312@live.cn (J.T.); chinj_cheung@163.com (W.Z.); hnzeric@hotmail.com (H.Z.); cxt0804@163.com (X.C.); yahui.gao@scau.edu.cn (Y.G.); yxl@scau.edu.cn (X.Y.); 2 State Key Laboratory of Biocontrol, School of Life Sciences, Sun Yat-sen University, Guangzhou 510275, China; liuxh8@mail.sysu.edu.cn

**Keywords:** Southeast Asian pig, gene flow, PigGTEx, multi-omics database, selection signature, gene function

## Abstract

The domestic pig (*Sus scrofa*) and its subfamilies have experienced long-term and extensive gene flow, particularly in Southeast Asia. Here, we analyzed 236 pigs, focusing on Yunnan indigenous, European commercial, East Asian, and Southeast Asian breeds, using the Pig Genomics Reference Panel (PGRP v1) of Pig Genotype-Tissue Expression (PigGTEx) to investigate gene flow and associated complex traits by integrating multiple database resources. In this study, we discovered evidence of admixtures from European pigs into the genome of Yunnan indigenous pigs. Additionally, we hypothesized that a potential conceptual gene flow route that may have contributed to the genetic composition of the Diannan small-ear pig is a gene exchange from the Vietnamese pig. Based on the most stringent gene introgression scan using the *fd* statistic, we identified three specific loci on chromosome 8, ranging from 51.65 to 52.45 Mb, which exhibited strong signatures of selection and harbored the *NAF1*, *NPY1R*, and *NPY5R* genes. These genes are associated with complex traits, such as fat mass, immunity, and litter weight, in pigs, as supported by multiple bio-functionalization databases. We utilized multiple databases to explore the potential dynamics of genetic exchange in Southeast Asian pig populations and elucidated specific gene functionalities.

## 1. Introduction

Genetic introgression is a fundamental evolutionary process that plays a crucial role in shaping the genetic diversity of species. It serves as the raw material upon which the genetic variation within a species is built [1,2,3,4]. Both naturally occurring and artificially mediated genetic introgressions among pig breeds/populations have the potential to significantly impact and reshape a wide range of phenotypes [5,6,7,8,9,10,11,12,13]. The Large White pig breed is renowned for its prolificacy, meat quality, and adaptability to various environments. Its genetic makeup reflects the influence of genetic introgression, as it carries a combination of genetic variations inherited from both European and Asian pig populations [6,7]. As a prominent center of pig domestication in Asia, it has been suggested that China was home to multiple pig domestication sites. These sites include regions, such as the upstream area of the Mekong River, the highlands of the Tibetan plateau, and the upstream and middle-to-downstream areas of the Yangtze River [10]. By analyzing the maternal genetic lineage of pigs through mitochondrial DNA analysis, researchers have identified genetic signatures that indicate early domestication events in the Yellow River basin [11]. According to previous research, Chinese porcine breeds were believed to have directly descended from local stocks of pigs that were domesticated during the Neolithic period. Specifically, the indigenous pig breeds, such as Erhualian, Meishan, and Jiangquhai pigs, which originated from the lower reaches of the Yangtze River, are considered to have maintained a closer lineage inheritance to ancient pigs compared to other regional breeds [12]. Indeed, both the selection and genetic admixture from exotic breeds have significant roles in the breeding process of domestic pigs, contributing to their genetic architecture and improvements in important economic traits. Several lines of evidence support this: During the Industrial Revolution, the presence of shared haplotypes between Meishan and Duroc pig breeds further supported the notion of Asian-derived introgression and the specific contribution of Meishan pigs to increasing sow fertility in European pigs [7,13,14,15]; the detection of more than a quarter of the genetic components of European commercial pigs in current Laiwu pig populations indicates large-scale interspecific crosses and gene introgression; these processes have been instrumental in incorporating genetic material from European breeds and improving important economic traits in Laiwu pigs [16]. These findings collectively emphasize the role of selection and genetic admixture in pig breeding, showcasing how intentional hybridization and introgression can lead to the enhancement of desired traits and improvements in economic performance in pig populations [7,9,13,14,15,16].

Yunnan Province, situated in Southwest China, benefits from its unique geographical location and diverse climate, which encompasses various ecosystems. This region has a rich history of pig breeding and is known for its abundant pig genetic resources. Additionally, Yunnan is home to several distinct and exceptional indigenous pig breeds that have adapted to the local environment [17,18]. Some notable examples include Saba, Diannan small-ear, Baoshan, Gaoligongshan, Diqing Tibetan, Dahe, Guangming small-ear, Lijiang, and Zhaotong pigs [19]. A previous study on Yunnan indigenous pigs revealed that there were at least two distinct maternal domestication events based on the genetic variation observed in the mitochondrial DNA D-loop region [20]. Additionally, as Yunnan is an important migration route from Southeast Asia into China, the region is home to hybrid pigs with diverse lineages [20,21,22]. Moreover, during the early Pliocene, Eurasian wild boars and other sister species emerged in Southeast Asia, leading to frequent hybridization events and the coexistence of different suid species. These hybridization events facilitated gene flow, resulting in the transfer of both adaptive and maladaptive alleles among pig populations and subfamilies [23]. This process contributes to the genetic diversity and adaptive potential of pigs in Yunnan [24]. Understanding the genetic history and hybridization events in Yunnan’s pig populations provides valuable insights into their genetic diversity, adaptation, and potential for future breeding and conservation efforts. It highlights the dynamic nature of genetic interactions and the ongoing evolutionary processes that shape pig populations in the region.

The advancement of next-generation sequencing technology and the availability of re-sequencing databases have greatly facilitated whole-genome sequencing (WGS) analysis studies. These studies have become increasingly important in various areas of research, including the origin, evolution, domestication, demographic history, ancient genomics, selection signatures, migration, and introgression events [25,26]. By utilizing WGS analysis and establishing multi-omics databases, researchers can gain valuable insights into the biological significance of genetic information, particularly considering that many genes exhibit high homology across different species. This approach allows for the exploration of shared genetic features and functional elements across organisms, enabling comparative genomics studies [27,28,29,30]. For instance, the Pig Genotype-Tissue Expression (PigGTEx) project, which is a part of the Farm animal Genotype-Tissue Expression (FarmGTEx) project, utilized WGS analysis to investigate gene expression and gene regulatory effects in pigs. Recent findings from the PigGTEx project have revealed that the tissue specificity of gene expression and gene regulatory effects are often conserved between pigs and humans. The public resources provided by PigGTEx offer a valuable platform for studying the evolution, genetic introgression, gene flow, complex traits, and molecular regulation between Yunnan pigs and pig populations in neighboring countries or regions within Southeast Asia [27,30]. They enable us to investigate the genetic and molecular mechanisms underlying important traits and the impact of genetic exchange and introgression events on pig populations in Yunnan and Southeast Asia as a whole [27,30].

To address these critical questions related to introgression from Southeast Asian pigs to Yunnan indigenous pigs, we selected five indigenous pig breeds from Yunnan due to their slow growth, low fat content, superior meat quality, resilience to coarse feed, strong stress resistance, and adaptability, particularly to high-altitude climates. These data were analyzed alongside genomic data from European commercial pigs, as well as East Asian and other Southeast Asian pig populations in the Pig Genomics Reference Panel (PGRP v1) of PigGTEx. The combined dataset allowed for the investigation of population structure, differentiation, and gene flow among these pig populations. Of particular importance, the study identified key introgressed regions and regions under selection. These regions represent areas of the genome where genetic material from Southeast Asian pigs has been introgressed into Chinese pig populations. The identification of these regions provides valuable insights into the specific genes and genetic variants that have been transferred between pig populations, shedding light on the genetic basis of important traits and the impact of introgression on the genetic diversity and adaptation of Yunnan indigenous pigs. Overall, the analysis of whole-genome data and the identification of introgressed and selection regions have contributed to a deeper understanding of gene introgression from Southeast Asian pigs into Yunnan indigenous pigs. These findings provide new insights into the genetic exchanges and evolutionary dynamics between pig populations in Southeast Asia and China, enhancing our knowledge of pig genetic resources, breed development, and adaptation in the region, and the potential functional implications of these genetic changes in traits related to pig production and morphology.

## 2. Results

### 2.1. Genetic Relationships and Population Structure of Yunnan Indigenous Pigs

**Figure 1:**
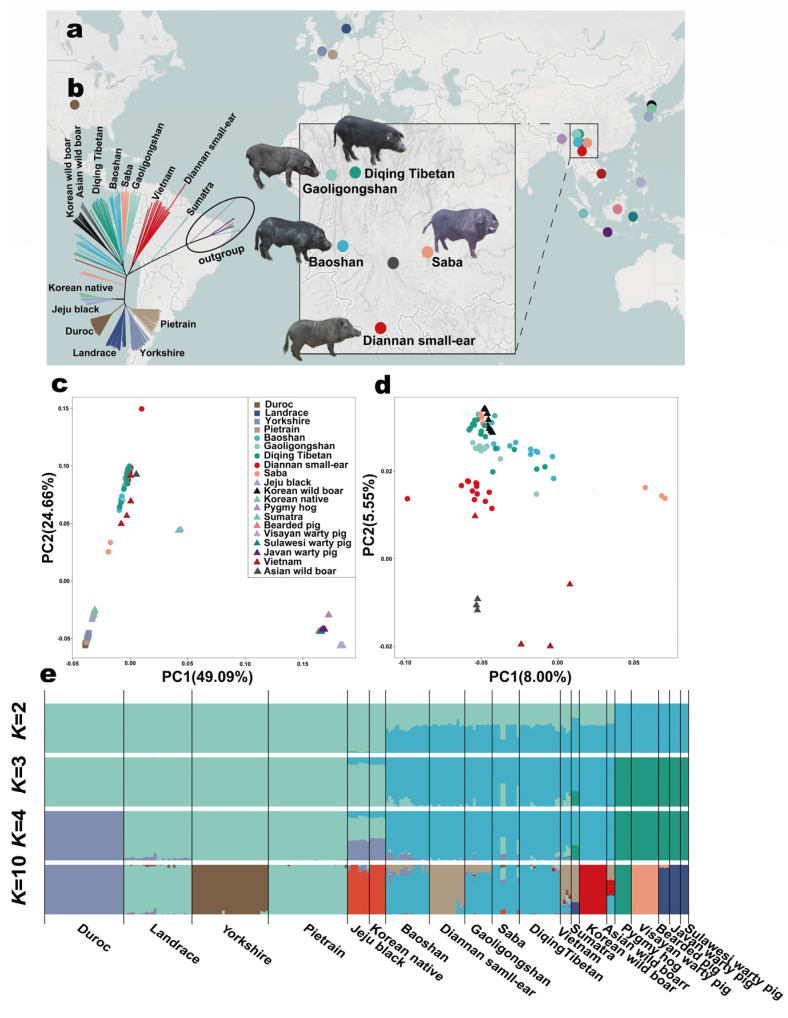
Geographic distribution and population structure. (**a**) Sampling and geographic sites of Yunnan, European, East Asian, and other Southeast Asian pig breeds/populations. The color scheme reflects the mirroring of geographic sites and population structure results (refer to the legend in (**c**)). (**b**) The Neighbour-Joining (NJ) phylogenetic tree represents the genetic relationships among 236 pigs based on whole−genome sequencing data. (**c**) Principal component analysis (PCA) results of 236 individuals from 20 pig breeds/populations on the first two PCs. (**d**) Further PCA analysis excluding European, Korean, Sumatra and Island Southeast Asian pigs. (**e**) The ancestry compositions of European and Southeast Asian pigs using Admixture with the assumed number of ancestries from *K* = 2 to *K* = 4 and *K* = 10, which had the lowest cross−validation error (cv error), different colors represent different ancestral components.

To clarify the population genetic structure among Yunnan indigenous pigs, commercial pigs, East Asian, and other Southeast Asian pigs, we analyzed the 236 individuals from PGRP (v1) of PigGTEx (Figure 1a, Supplementary Table S1). In Neighbour-Joining (NJ) tree analysis using autosomal SNPs, all commercial pigs from the same breeds clustered together, and Diannan small-ear pigs formed a separate clade, distinct from other Yunnan indigenous pig breeds. However, the other four Yunnan pig breeds exhibited mixed patterns in the tree (Figure 1b). Korean pigs (Jeju black and Korean native) were genetically closer to European commercial pigs. However, Korean wild boars and Asian wild boars were genetically closer to Yunnan pigs (Figure 1b). The analysis revealed three main lineages (Lineage I, II, and III), with nine sub-branches (A to I) in Lineage III. These lineages and sub-branches distinguish European commercial pigs and East Asian pigs from Southeast Asian pigs, including Yunnan pigs. Diannan small-ear and Vietnam pigs defined a separate clade (Lineage II), indicating less genetic differentiation and similar genetic makeup in these populations (Supplementary Figure S1). Principal component analysis (PCA) analyses showed that PC1 explained 49.09% of the total eigenvalue, which separated domestic pigs, Asian wild boars, and Korean wild boars from Southeast Asian domestic pigs and Island Southeast Asian pigs. PC2 explained 24.66% of the total variation, further separating European, East Asian, and other Southeast Asian pigs (Figure 1c). When excluding European, Korean, Sumatra, and Island Southeast Asian wild pigs, the PCA analyses showed that Diannan small-ear pigs were divided into Yunnan pigs and had a closer genetic relationship with Vietnam and Asian wild boars. There was a mixed pattern observed between Korean wild boars and other Yunnan pig breeds (Figure 1d). Admixture analysis was performed to estimate ancestral components (Figure 1e, Supplementary Figure S2). When *K* = 2, Asian and European pigs represented two different ancestral components, and the Korean pig population had a larger proportion of admixture with European pig lineages than other Southwest Chinese pigs. Some Southwest Chinese pig breeds (Baoshan and Saba) also displayed significant admixture with European breeds. When *K* = 3, the Island Southeast Asian pig was separated from the Southeast Asian pig. Island Southeast Asian pigs’ ancestry was detected in Sumatra pigs. Up to *K* = 4, Duroc formed an independent lineage, and Korean and Southwest Chinese pigs showed admixture with Duroc relationship patterns (Figure 1e). Notably, at *K* = 10, which had the lowest cross−validation error (cv error) (Supplementary Table S2), the Southeast Asian domestic pig genomes showed varying proportions of European ancestry. Diannan small-ear pigs exhibited unique genetic information among Yunnan pigs and shared genetic ancestry with Baoshan, Gaoligongshan, Vietnam, Sumatra, and Asian wild boars. Additionally, European, Diannan small-ear, Korean wild, and other Yunnan domestic pig ancestries were still present in Vietnam pigs (Figure 1e). These results were consistent with the NJ tree and PCA analysis (Figure 1b–e).

### 2.2. Genetic Differentiation and Gene Flow Analyses

**Figure 2:**
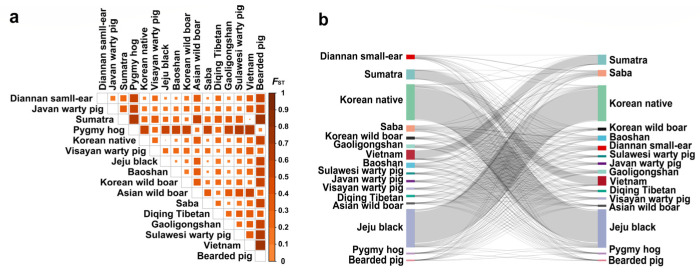
Genetic relationship among 16 pig breeds/populations. (**a**) Heat-map of genetic differentiation among 16 pig breeds/populations based on the fixation index (*F*_ST_) values. Dark colors and larger squares represent higher *F*_ST_ values. (**b**) Gene flow among 16 pig breeds/populations based on the gene flow levels (Nm).

The fixation index (*F*_ST_) values among the 16 pig breeds/populations ranged from 0.001 to 0.726, indicating varying degrees of genetic differentiation (Figure 2a, Supplementary Table S3). In Yunnan pigs, the estimates of *F*_ST_ values showed weak (0 < *F*_ST_ < 0.05) or moderate (0.05 < *F*_ST_ < 0.15) genetic differentiation between Yunnan pig populations, excluding Diqing Tibetan pigs; however, there was high (0.15 < *F*_ST_ < 0.25) genetic differentiation between Diqing Tibetan and the other four Yunnan pigs. In other pig populations, there was weak (0 < *F*_ST_ < 0.05) genetic differentiation between Jeju black and Korean native (0.001) as well as Vietnam and Sumatra (0.004); in addition, this was also seen among Jeju black and four Yunnan pigs (Baoshan, Diannan small-ear, Gaoligonghsan, and Saba) (0.021 < *F*_ST_ < 0.045). Furthermore, Diqing Tibetan has moderate (0.05 < *F*_ST_ < 0.15) genetic differentiation between Visayan warty pigs (0.099) and Asian wild boars (0.130). The gene flow levels (Nm) among 16 pig breeds/populations were from 0.094 to 249.750 (Figure 2b, Supplementary Table S4), which indicated that there was extensive gene flow among pig species. A particularly high gene flow was observed between Jeju black and Korean native (Nm = 249.750) and between Vietnam and Sumatra (Nm = 62.250) (Figure 2b).

### 2.3. Migration and Introgression Events Analysis

**Figure 3:**
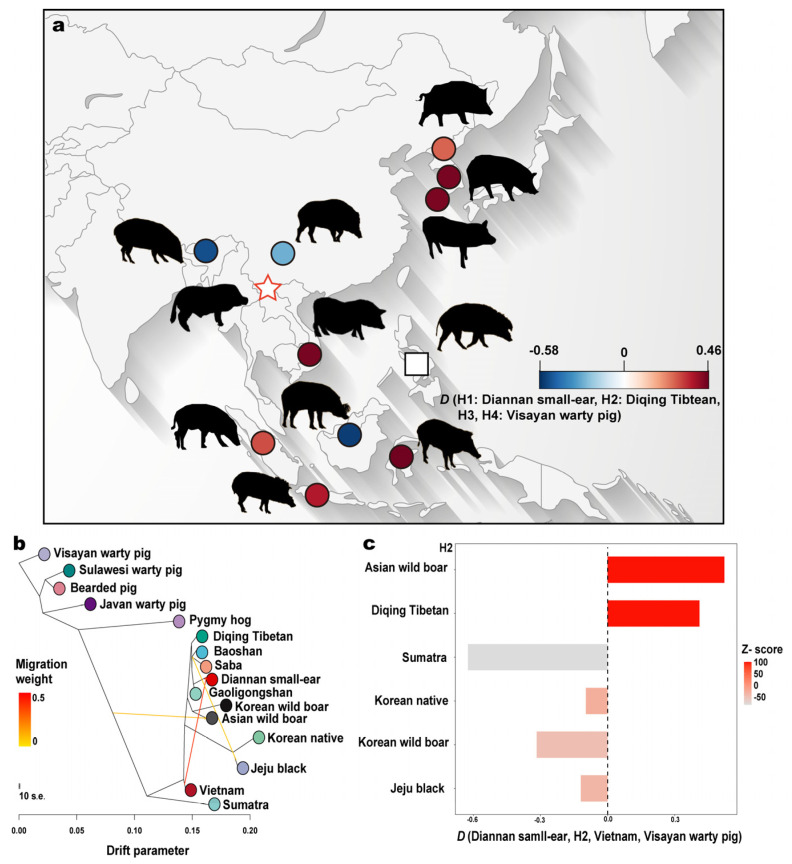
Migration and introgression events of 16 pig breeds/populations. (**a**) A heat-map of *D*-statistic testing for the differential affinity between H3, H1: Diannan small-ear (star in the map) and H2: Diqing Tibetan, where H3 represents the individual or population of wild and domestic pigs located in the map. H4: Visayan warty pig (square in the map) represents the outgroup. Negative *D* values indicate that H3 shares more derived alleles with H2 than H1. Positive *D* values indicate that H3 shares more derived alleles with H1 than with H2. Colors closer to warm indicate positive values of the *D*-statistics, while colors closer to cool indicate negative values. The conceptual graphs of pigs were acquired from ©Brent Huffman (www.ultimateungulate.com, accessed on 5 December 2022). (**b**) TreeMix analysis revealing gene flow and migration events among 16 pig breeds/populations. Visayan warty pig (*Sus Cebifrons*) was used as an outgroup to root the trees. (**c**) Allele sharing between Vietnam and Diannan small-ear, or Vietnam and H2, H2 represents the Asian wild boar, Diqing Tibetan, Sumatra, Korean native, Korean wild boar, and Jeju black. Visayan warty pig (*Sus Cebifrons*) represents the outgroup.

Treemix and *D*-statistics reveal significant signals of allele sharing with Diannan small-ear and Vietnam pigs (Figure 3a–c, Supplementary Figures S3–S5). When considering three populations (m = 3), the analysis revealed substantial gene flow from Vietnam into Diannan small-ear pigs and from Jeju black pigs into Yunnan pigs (Baoshan and Saba) (Figure 3b). Similarly, when considering five populations (m = 5), significant gene flow was observed from Vietnam into Diannan small-ear pigs, Jeju black into Yunnan pigs (Baoshan and Saba), Jeju black and Korean native pigs into Korea wild and Saba pigs, and Javan warty pigs into Sumatra pigs (Supplementary Figure S3). Treemix also confirmed gene flow from Yunnan pigs (Saba) to Jeju black and Korean native pigs (Supplementary Figure S3). *D*-statistics were used to investigate gene flow in more detail. Two conceptual routes of gene flow to Diannan small-ear pigs in Southeast Asia were identified (Figure 3a,c, Supplementary Figures S4 and S5). One route involved gene flow from Korean populations (Korean native, Jeju black, and Korean wild boar) to Diannan small-ear pigs. The other route involved gene flow from Sulawesi warty pigs, Visayan warty pigs, Sumatra, and Vietnam into Diannan small-ear pigs (Figure 3a). Calculations of *D*-statistics (Diannan small-ear, H2; Vietnam, Visayan warty pig) yielded significant positive values (*Z*-scores were 100, *Z*-scores were a standard score, it is the process of subtracting a number from the mean and then dividing it by the standard deviation), indicating strong gene flow between Vietnam and Diannan small-ear pigs. Additionally, low introgression percentages of Diannan small-ear pigs to Diqing Tibetan pigs and Asian wild boars were also observed (Supplementary Table S5 and Supplementary Figures S4 and S5).

### 2.4. Multiple-Gene Introgression of Vietnam to Diannan Small-Ear

**Figure 4:**
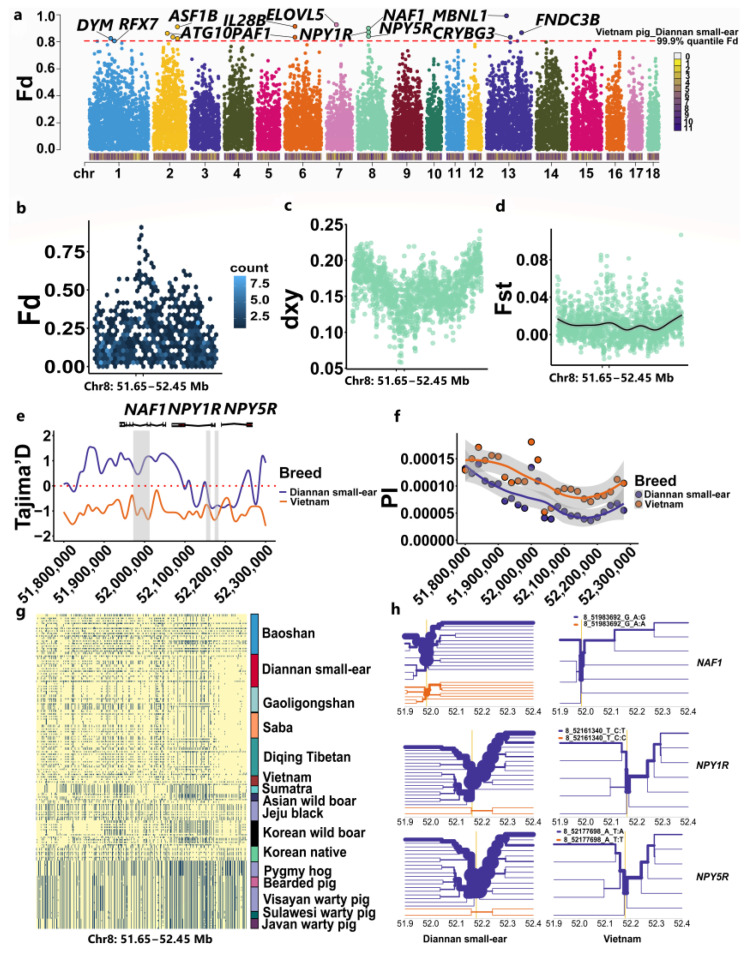
Multiple-gene introgression scan from Vietnam to Diannan small-ear. (**a**) Manhattan plot of *fd* values estimated for 100 kb windows, estimated from called genotypes using the four pig populations with Diannan small-ear pigs as H2, Baohsan pigs as H1, which is the sister group of Diannan small-ear, Vietnam pigs as H3, and the *Sus Verrucosus* as H4 (outgroup). We selected 99.9% quartile *fd* as the most apparent infiltration area (red dotted line), different colors represent corresponding chromosomes. (**b**) Distribution of the *fd* values plotted in chromosome 8, different colors represent enriched plots. (**c**) Plot of Average number of nucleotide differences (dxy)within Diannan small-ear and Vietnam pigs in chromosome 8. (**d**) Plot of *F*_ST_ within Diannan small-ear and Vietnam pigs in chromosome 8. Line and (narrow) shaded areas are fitted values and 95% confidence limits from a linear regression. (**e**) Tajima’s D plots at *NAF1*, *NPY1R* and *NPY5R* gene region, these gene bodies were sourced from the Ensembl database (https://asia.ensembl.org/, accessed on 25 May 2023). (**f**) Nucleotide diversity (PI) plots at the 51.65 Mb-52.45 Mb of chromosome 8 region. Line and (narrow) shaded area are fitted values and 95% confidence limits from a linear regression. (**g**) Degree of haplotype sharing across pig populations at the 51.65 Mb-52.45 Mb of chromosome 8 region. The major allele at each SNP position is colored in yellow. (**h**) Bifurcation diagram for extended haplotypes in *NAF1* (chr8_51983692_G_A), *NPY1R* (chr8_52161340_T_C) and *NPY5R* (chr8_52177698_A_T) gene region. Left, Diannan small-ear pigs; right, Vietnam pigs.

We conducted an analysis using Treemix and *D*-statistics (Figure 3a–c, Supplementary Table S5), which revealed strong evidence of introgression from Vietnam into Diannan small-ear pigs. The *fd* statistic was used to measure the extent of shared genomic regions resulting from this introgression. A Manhattan plot and the number of SNPs within a 0.01 Mb window size were used to visualize the genome-wide distribution and identify introgression regions (Figure 4a, Supplementary Figure S6). The top 0.01% windows with the highest *fd* signals are shown in Figure 4a. The presence of high *fd* values indicated a substantial transfer of genetic material from Vietnam to Diannan small-ear pigs. These windows exhibited exceptionally high *fd* values and low *F*_ST_ and Average number of nucleotide differences (dxy) values between Vietnam and Diannan small-ear pigs compared to the genomic averages, particularly in chromosome 8 (Figure 4b–d). We selected the top outlier window (Chr 8:51.65 Mb-52.45 Mb) as the most promising candidate for adaptive introgression based on the available data. Within the identified introgression regions, three genes stood out: *NAF1*, *NPY1R*, and *NPY5R*. In addition, analysis using Tajima’s D test also showed significant departure from neutrality and indicated the selective maintenance of alleles within the Vietnam population compared to the Diannan small-ear breed. Negative values of Tajima’s D indicate an excess of rare variation, and we observed a rapid drop in Tajima’s D value within regions of the candidate gene under selection in Vietnam. And the Tajima’s D for the *NAF1* gene region in Diannan small-ear pigs is greater than 0, suggesting this region experiences a bottleneck effect or balancing selection. Analyzing these genes revealed that Diannan small-ear pigs had lower nucleotide diversity (PI) and differential Tajima’s D values, suggesting genetic changes associated with selective pressures (Figure 4e,f, Supplementary Table S6). The SNPs of *NPY1R* and *NPY5R* also showed high levels of linkage disequilibrium (LD) (Supplementary Figure S7). The identified Quantitative Trait Locis (QTLs) in the selection regions were associated with carcass weight, ham weight, loin muscle area, and teat number (Supplementary Table S7). Furthermore, we analyzed the haplotype diversity pattern of this window and the three crucial genes across 16 pig breeds/populations (Figure 4g). The results showed a significant difference between Yunnan indigenous pigs and other Southeast Asian pigs, as well as a consistent haplotype diversity pattern between Vietnam and Diannan small-ear pigs (Figure 4g, Supplementary Figure S8), indicating shared genetic ancestry and potential introgression events. Additionally, extended haplotype homozygosity tests confirmed that the haplotype carrying the alleles of *NAF1* (chr8_51983692_G), *NPY1R* (chr8_52161340_T), and *NPY5R* (chr8_52177698_A) extended further than alternative haplotypes between Vietnam and Diannan small-ear pigs (Figure 4h), emphasizing the selective pressure and potential adaptive value of these alleles in Diannan small-ear and Vietnam pigs.

### 2.5. Enrichment Analysis of Candidate Genes in Introgression Genes

Annotations of the highlighted introgression genes revealed functions that may be associated with important traits, early organ development (*DYM* and *RFX7*), diseases (*ASF1B*, *ATG10*, *PAF1* and *IL28B*), fatty deposits (*ELOVL5*), body height (*NPY1R*, *NPY5R*, *MBNL1* and *FNDC3B*), immunity (*NAF1*), and visual perception (*CRYBG3*) (Supplementary Table S6). Among them, *ELOVL5* was associated with the traits of tail fat deposition and the regulation of hepatic triglyceride catabolism [31,32]. *MBNL1* was associated with the traits of body size and impaired movement [33]. *ATG10* played an important role in the carcinogenesis of multiple organs [34,35]. To further elucidate the genetic mechanisms related to the introgression genes, we identified the vital 98 Kyoto Encyclopedia of Genes and Genomes (KEGG) and 261 Gene Ontology (GO) pathways in our enrichment results. As for functional enrichment analysis (Supplementary Table S8 and Supplementary Figure S9), the KEGG pathway had three significant functions, called “Neuroactive ligand-receptor interaction”, “Regulation of lipolysis in adipocytes”, and “cAMP (cyclic adenosine monophosphate) signaling pathway” (*p*-value < 0.01). In the ssc04080 pathway, which played an important role in the regulation of neuron function through modulating transcription factors and gene expression [36], we identified two genes (*NPY1R* and *PRKACA*) enriched in functions related to the regulation of lipolysis in adipocytes. Lipids have the highest energy density, which provides a great advantage for their energy storage and consumption, and some lipid metabolites act as signal mediators to regulate immune and metabolic homeostasis [37]. The cAMP signaling pathway is widely distributed in organisms, such as mammalian cells, plant cells, and microorganisms and is involved in the regulation of several physiological processes, such as growth, differentiation, metabolism, immunity, and neural function, which may contribute to growth, development, disease resistance, and environmental adaptation in domestic pigs [38]. GO terms were particularly enriched in terms of organ development and receptor activity (“outflow tract morphogenesis, GO:0003151”, “peptide YY receptor activity, GO:0001601”, and “acetylcholine receptor activity, GO:0015464”). Two genes (*PLD1* and *LIPG*) were enriched on pathways associated with the lipid catabolic process (GO:0016042), which underlined the stronger lipolysis and conversion metabolism in Southwest China pigs. In addition, some significant GO terms (transcription elongation from RNA polymerase II promoter, GO:0006368, RNA splicing, GO:0008380 and cytosolic small ribosomal subunit, GO:0060079) related to important biological process in gene expression were detected, which play an important role in genetic regulation within organisms.

### 2.6. Cell Expression of the NAF1 Gene in Humans

**Figure 5:**
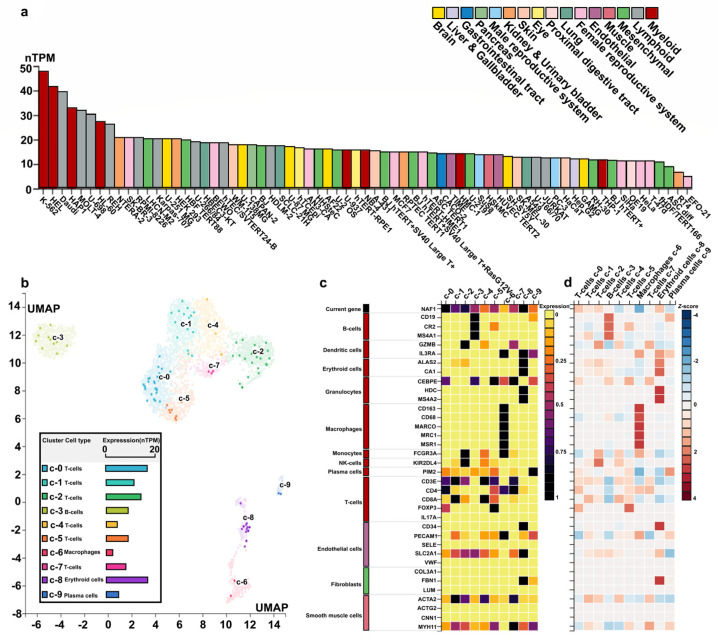
Cell expression of the *NAF1* in humans. (**a**) RNA cell line category. According to their RNA expression levels across the panel of cell lines. Cell lines are ordered by descending RNA expression order. (**b**) RNA expression in the single-cell-type clusters identified in bone marrow tissue visualized by a UMAP (Uniform Manifold Approximation and Projection) plot and a bar chart. Colored according to cell-type group. Scatter plot, all cells color scale % of max. nTPM (normalized transcripts per million): transcripts per kilobase of exon model per million mapped reads. (**c**) The heatmap in this section shows the expression of the *NAF1* and well-known cell-type markers in the different single-cell-type clusters of bone marrow. The panel on the left shows which cell type each marker is associated with. Color coding is based on cell-type groups, each consisting of cell types with functional features in common. (**d**) The heatmap of *Z*−score in this section shows expression of the *NAF1* and well-known cell-type markers in the different single-cell-type clusters of bone marrow.

Expression analysis of the *NAF1* gene in human cells revealed its presence in both the nucleoplasm and cytosol (Supplementary Figure S10). Additionally, when analyzing RNA expression in various cell lines, it was found that the *NAF1* gene was fully expressed in myeloid cell lines, such as K-562, HEL, and HAP1, as well as in lymphoid cell lines, including Daudi, MOLT-4, and U-698 (Figure 5a). Notably, all of these cell lines belong to the cancer cell category. Based on this information, two specific single-cell types, namely bone marrow and lymph node cells, were selected for further analysis (Figure 5b). Within the bone marrow cell population, the *NAF1* gene was found to be widely expressed in various subcellular cells, as indicated by different colors (Figure 5b). Particularly high expression levels of *NAF1* were observed in erythroid cells (16.8 nYPM) belonging to the c-8 cluster, as well as in T cells (16.5 nYPM) of the c-0 cluster. To compare the expression of the *NAF1* gene with well-known cell-type marker genes in different single-cell-type clusters within the tissue, further analysis was performed (Figure 5c). In the T cells of the c-0 cluster, both the *NAF1* and *CD4* genes were highly expressed, with *Z*-scores of 1.11 and 1.18, respectively, indicating their potential co-occurrence or functional relationship within this cell type (Figure 5c,d). Furthermore, in erythroid cells of the c-8 cluster, the *NAF1* gene exhibited high expression along with the *ALAS2* and *CA1* genes (Figure 5c). The corresponding *Z*-scores for *NAF1*, *ALAS2*, and *CA1* were 1.14, 2.03, and 2.37, respectively (Figure 5d). Moreover, significant expression of the *NAF1* gene was observed in lymph nodes. Particularly, B cells in the c-0 and c-5 clusters showed higher expression levels of *NAF1* (36.6 nYPM and 42.3 nYPM, respectively), along with T cells in the c-1 cluster (36.1 nYPM) (Supplementary Figure S11). Overall, these findings indicate that the *NAF1* gene is expressed in several important immune organs, such as the bone marrow and lymph nodes. This expression pattern suggests that the *NAF1* gene plays a significant role in disease resistance and immune system function in mammals.

### 2.7. PigGTEx, Human PheWAS and Mouse Knockouts of NPY5R Gene

**Figure 6:**
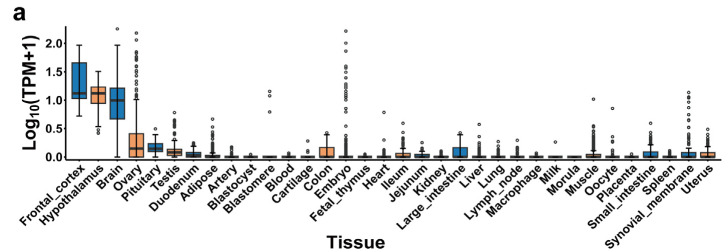

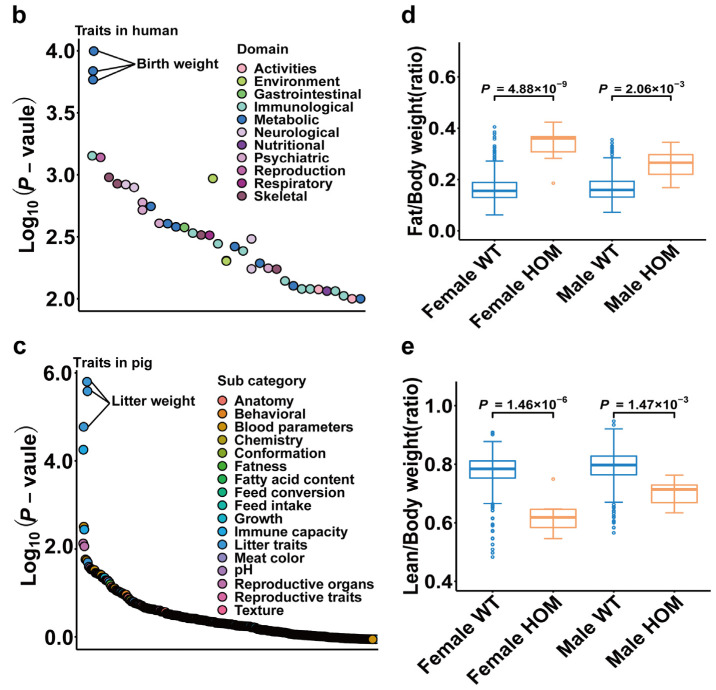
PigGTEx, human PheWAS and mouse knockouts of *NPY5R*. (**a**) Bulk tissue gene expression for *NPY5R* gene in pigs by using the PigGTEx (Pig Genotype-Tissue Expression) database, each black dot represents the tissue expression of a sample. (**b**) *NPY5R* gene in human PheWAS (Phenome-wide association studies). For SNPs, 0.01 is the maximum *p*-value. Different colors are based on phenotype type groups, each consisting of phenotypes with functional features in common. (**c**) *NPY5R* gene in pig GWAS (Genome-Wide Association Studies) of complex traits. Different colors are based on phenotype type groups, each consisting of phenotypes with functional features in common. (**d**) The graph page shows an IMPC (international mouse phenotype Consortium) comparison of male and female *NPY5R* knockout and normal mice with fat/body weight (ratio). (**e**) The graph page shows an IMPC comparison of male and female *NPY5R* knockout and normal mice with lean/body weight (ratio). Each point represents two variables in the dataset, the value for the measured parameter (vertical axis) in a given mouse and the date on which the parameter was measured (horizontal axis).

The phenotypic results provide insights into the function of several genes, including *NPY5R* (Supplementary Tables S9 and S10 and Supplementary Figures S12–S15). In PigGTEx analysis, the *NPY5R* gene exhibited tissue-specific expression in the brain and ovary, particularly in the frontal cortex and hypothalamus (Figure 6a). In the *NPY5R* gene of human PheWAS, the *NPY5R* gene was analyzed in association with human phenotypes. A criterion was set (*p*-value < 0.01, sample size > 5000) to filter GWAS data (Supplementary Table S9). Three points with high significance were identified in the scatter plots, specifically associated with birth weight (Figure 6b, Supplementary Table S9). In the *NPY5R* gene of pig complex traits, the enrichment of litter weight in relation to the *NPY5R* gene suggests its involvement in reproductive traits in pigs (Figure 6c, Supplementary Table S10). In mouse knockouts of *NPY5R* gene, a phenotypic assay of body composition was performed on 1305 mice. The charts show the results of measuring fat and lean mass in 7 female and 8 male mutants compared to 671 female and 619 male controls (Figure 6d,e). The mutants are for the *NPY5R*^em1(IMPC)H^ allele. Based on the Linear Mixed Model framework, not including weight, the *p*-value of the female genotype was 4.88 × 10^−9^ between wild-type (WT) and homozygous (HOM) mice in fat/body weight (ratio), and the *p*-value of the male genotype was 2.06 × 10^−3^ (Figure 6d). Similarly, only considering the lean/body weight (ratio), the *p*-value of the female genotype was 1.46 × 10^−6^ between WT and HOM mice, and the *p*-value of the male genotype was 1.47 × 10^−3^ (Figure 6e). The findings from the mouse knockout study indicate that the *NPY5R* gene plays a crucial role in regulating body composition, specifically fat and lean mass. Knocking out the *NPY5R* gene resulted in a significant increase in the total body fat amount and a decrease in lean body mass in both male and female mice. These results suggest that the *NPY5R* gene is involved in regulating energy balance, metabolism, and body composition.

## 3. Discussion

There are rich genetic resources of pig breeds in the world because of genetic introgression and hybridization between different pig breeds [5,6,7,8,9]. Contrary to previous studies, despite the unique geographical location and complex climate system of Yunnan province in Southern China, the genomic and genetic introgression of indigenous pigs in Yunnan is insufficient. In this study, we conducted genome-level analyses of Yunnan indigenous pigs, commercial pigs, and other East Asian and Southeast Asian pig samples from the PGRP (v1) to gain insights into population structure, differentiation, gene flow, adaptive introgression, signature selection, and gene function. In our study, European commercial pigs, including Duroc, Landrace, Yorkshire, and Pietrain, formed a distinct group separate from Yunnan pig breeds in various genetic analyses, such as the NJ tree, Admixture, and PCA (Figure 1b–e). This finding is consistent with previous studies on European commercial pigs and Chinese native pigs [5,6,7,9,13,14,15,16]. However, minor European commercial pig ancestry was identified in all Yunnan indigenous pig breeds. Additionally, Diannan small-ear pig ancestry was detected in the genomes of other Yunnan pig breeds, except Saba pigs, indicating that human-mediated admixture has had specific effects on Yunnan pigs [5,6,7,9,13,14,15,16]. It has been well documented that introgression has occurred between Western commercial pigs and Chinese indigenous breeds, as well as between excellent Chinese pigs and European breeds [5,6,7,9,13,14,15,16]. Notably, Diannan small-ear pigs showed genomic uniqueness compared to commercial pigs and other Yunnan indigenous pigs, likely due to their geographical isolation (Figure 1b–e). To further investigate the genetic relationships, fixation index, gene flow, and introgression events affecting Diannan small-ear pigs, additional East Asian and Southeast Asian pig samples were included in the analysis (Figure 2a,b and Figure 3a–c). The results revealed a close genetic distance between Yunnan and Korean pigs (Jeju black, Korean native, and Korean wild), with the exception of Diqing Tibetan pigs. Surprisingly, Diqing Tibetan pigs exhibited significant genetic distance from other Yunnan indigenous pigs while showing closer genetic proximity to Sulawesi warty pigs and Asian wild boars (Figure 2a,b). This suggests earlier mixing events in the history of these pig populations. Vietnam and Sumatra pigs showed a closer relationship with Korean wild pigs, while Jeju black populations exhibited signals of admixture with Korean native pigs, aligning with previous studies (Figure 2a,b, Supplementary Tables S3 and S4) [10]. These findings support the hypothesis that Yunnan may be a domestication center or an origin location for Tibetan pigs [39]. In addition, we found distinct genetic differentiation between Vietnamese pigs and Diannan small-ear pigs (*F*_ST_ = 0.305). This differentiation might primarily stem from the Vietnamese pig individual in the Central-Western pig NJ tree, harboring a significant number of alleles from Western commercial pig breeds. And the *F*_ST_ value between Jeju black and Korean native pigs is only 0.001, indicating minimal genetic differentiation, likely originating from the same breed/population (Supplementary Table S3). Treemix and *D*-statistics analyses were conducted to further explore admixture and migration events in Southeast Asian pigs (Figure 3a–c), revealing significant migration signals from Vietnam pigs to Diannan small-ear pigs (Figure 3b, Supplementary Figure S3). Treemix from Jeju black and Korean native pigs to Yunnan pigs was also identified (Figure 3b), but most pigs in Korea have been crossed with European pig breeds and, thus, are not true representatives of Korean native and Jeju black pigs [40]. Therefore, our findings suggest that there has been genetic introgression of Western commercial pig breeds into Yunnan pigs. Similarly, Korean pigs show significant ancestry originating from Western commercial pig breeds. Previous studies on the genetic variation in pigs through D-loop analysis of maternal lineage supported the closer relationship between Korean wild boars and South China pigs, as well as the similarity between Vietnam pigs and domestic pigs from the Yellow River Basin in North China [10,11,41]. These patterns are a result of complex and frequent historical human migrations and human-mediated importation of good bloodlines among Vietnamese, European commercial, and Southwest Chinese pig populations. This study supports the hypothesis that the Mekong River basin region, where Diannan small-ear and Vietnam pigs are located, is one of the origin points of domestic pigs [10]. The genetic information obtained from these populations provides further confirmation of the significance of this region in the domestication and diversification of pig breeds.

The emergence of various biological function databases will also provide a new foundation for a more precise interpretation of gene functions between species [27,28,29,30]. In our study, we discovered a significant signal of admixture from Vietnam pigs to Diannan small-ear pigs. As a result, we conducted further research to identify candidate genes that may be involved in introgression and multiple-gene introgression in Diannan small-ear pigs (Figure 3b,c and Figure 4a,b). One of the candidate regions (51.65 Mb–52.45 Mb) identified, harbored genes associated with immune function and body weight regulation (Figure 5a–d and Figure 6a–e, Supplementary Tables S9 and S10). Among these genes, the nuclear assembly factor 1 ribonucleoprotein (*NAF1*) is considered a housekeeping gene that is essential for maintaining basic cellular functions. It has also been implicated in regulating various cancer-related pathways [42,43]. Additionally, the neuropeptide Y 5 receptor (*NPY5R*) is expressed in the amygdala and hypothalamus of pigs and is involved in the regulation of food intake and energy expenditure [44,45]. We also compared these genes to human PheWAS and found significant enrichments. In the *NAF1* gene, associations were observed with black hair (natural, before greying), while the *NPY1R* gene showed enrichment with 25-Hydroxyvitamin D in humans (Supplementary Table S9 and Supplementary Figures S12 and S13). In pig complex traits, such as average daily gain, immunoglobulin G level, and litter weight, significant enrichments were found in the *NAF1*, *NPY1R*, and *NPY5R* gene regions, respectively (Figure 6c, Supplementary Table S10 and Supplementary Figures S14 and S15). Further analysis of pig bulk tissue transcriptomes revealed a high expression of *NPY5R* in the brain, specifically the frontal cortex and hypothalamus [27]. This finding aligns with results from human GTEx data (Supplementary Figure S16) [28], which also showed high expression of *NPY5R* in the brain. Previous studies have indicated that body mass index (BMI) and body weight regulation involve the genetic control of gene expression in the brain and adipose tissue, with expression quantitative trait loci (eQTLs) corresponding to tissue-specific genes [28,46]. The significant phenotypes associated with *NPY5R* in body weight, such as human birth weight in PheGWAS and mouse knockout experiments (Figure 6b–e), further support the importance of these genes in regulating body weight. In pig complex traits, litter weight showed the most significant association among 268 traits studied (Supplementary Table S10). Considering the long-term rigorous artificial selection and breeding of Diannan small-ear pigs, it is likely that these candidate genes, such as *NAF1*, *NPY1R* and *NPY5R*, play crucial roles in the productive performance of these pigs. Collectively, our research findings provide valuable insights into the potential genetic mechanisms underlying adaptive and multiple-gene introgression in Diannan small-ear pigs, particularly in relation to immune function and body weight regulation. The gene function identification in *NAF1*, *NPY1R*, and *NPY5R* helps us to better explain the complex traits in pigs. Subsequent utilization molecular marker-assisted selection techniques for these candidate genes in pig breeding to improve their disease resistance and economic efficiency. We also found that Chinese indigenous pigs were subjected to pedigree importation from European commercial pigs, so we suggest using live body and biotechnology for preservation in core preservation farms in order to maintain the complete genomic information of Chinese indigenous pigs.

Although some interesting findings were reported in this study, the limitations of the present study should not be neglected. (i) We acknowledge that our introgression scan was sensitive to the relatively small sample sizes (available Vietnam pig number = 3), which might not completely represent the populations and may have affected the Treemix, *D*-statistics, and *fd* statistic. (ii) Due to the complex early gene flow events in the Mekong River basin and the extensive hybridization and lineage introgression between wild boars and domestic pigs, especially manifesting in admixture of Vietnamese pigs, the gene flow we identified may exhibit a certain degree of sensitivity. Therefore, further analysis will require the introduction of more pure lineages for detailed investigation. (iii) Additionally, regarding the relevance of Korean pigs, including wild types, we do not have the corresponding samples or adequate data to provide a comprehensive evaluation of their genetic introgression. (iv) Although these introgression genes were highly homologous in humans, pigs, and mice, we also validated these genes using high-confidence data from multiple databases, but there is a lack of experimental validation in the model organism ontology of pigs. These limitations might impact the observations of this study, and the sample size and experimental validation should be increased in subsequent experimental analyses to improve the genetic potential of Southern Chinese pigs.

## 4. Materials and Methods

### 4.1. Newly Added Samples and Genotypes

Before sampling, we reviewed the pedigree records of farms to ensure the unrelatedness and representativeness of individuals within the breeds used for this study. We added newly sequenced high-depth whole-genome data from 64 Yunnan indigenous pigs (16 Baoshan, 10 Gaoligongshan, 15 Diqing Tibetan, 10 Saba and 13 Diannan small-ear) to the existing dataset of the PigGTEx. The analysis involved a total of 236 whole-genome sequences from the PGRP (v1) of the PigGTEx [27,30]. The dataset comprised 64 Yunnan indigenous pigs, 111 European commercial pigs, 14 East Asian pigs, including Jeju black and Korean native pigs, 34 Southeast Asian populations, including Pygmy hog (*P. salvinia*), Bearded pig (*S. Barbatus*), Visayan warty pig (*S. Cebifrons*), Sulawesi warty pig (*S. Celebensis*), Javan warty pig (*S. Verrucosus*), Sumatra, Vietnam, 10 Korean wild boars (*S. scrofa*), and 3 Asian wild boars from Southwest Chinese wild pigs (*S. scrofa*) (see Additional file 1: Table S1). Of particular interest, the Southwest Chinese wild pigs (Asian wild boar) included in this study were sampled from locations that closely border the Yunnan region in China. Geographical coordinates of the sampling sites were also obtained, providing valuable information about the origin and distribution of the samples. We used Trimmomatic (v0.39) to filter out the adaptors and low quality in 64 newly sequenced whole-genome data (commands: MINLEN:50 LEADING:20 TRAILING:20 SLIDINGWINDOW:5:20) [47]. We mapped clean reads to the pig reference genome using *Sus scrofa* release100 by using Burrows-Wheeler Aligner software (v0.7.5a) with default parameters [48]. Genome Analysis Toolkit (GATK, v4.1.4.1) was used to filter potential duplicate reads [49]. We also used GATK (v4.1.4.1) to call the SNP in PGRP (v1) of 236 individuals (QD > 2, MQ < 40, FS > 60, SOR > 3, MQRankSum < −12.5 and ReadPosRankSum < −8) [49]. To process the data, we filtered out SNPs with minor allele frequency (MAF) < 0.01 and/or call rate < 0.9 via BCFtools (v1.12) and phasing filtered variants using Beagle (v5.1); we also discarded all unmapped SNPs and those on sex chromosomes [50,51]. The SNP data from the autosomal chromosomes of the 236 samples of PGRP (v1) were combined into a single dataset for further analysis. This resulted in a final dataset containing 7,970,305 SNPs from the 18 autosomal chromosomes of the 236 samples.

### 4.2. Population Genetic Structure of Yunnan Indigenous Pigs

To investigate the population genetic structure of Yunnan indigenous pigs, several analyses were conducted using genome-wide SNPs. The following methods were employed: NJ tree analysis, PCA, and admixture analysis. For the construction of NJ trees, a pairwise genetic distance matrix was generated using the PLINK (v1.90) software [52]. The resulting trees were visualized using MEGA (v10.2.6) [53] and FigTree software (v1.4.4). PCA was performed using the PLINK (v1.90) software [52], and the output was visualized using ggplot2 in R (v4.2.1) [54]. To estimate the population genetic structure, ADMIXTURE (v1.3.0) [55] was employed. The analysis was performed considering various values of *K*, ranging from 2 to 21, representing different numbers of potential ancestral populations. The results were then visualized using R (v4.2.1) software [54].

### 4.3. Differentiation and Gene Flow Analyses

To assess population differentiation and gene flow among the 16 pig breeds/populations, *F*_ST_ and Nm were analyzed using genome-wide SNPs from the autosomes. The following methods and calculations were employed: For calculating *F*_ST_ values, the smartPCA program of the EIGENSOFT v5.0 software (with parameters numchrom: 18 and FSTonly: YES) was utilized [56]. To analyze gene flow levels among the 16 groups, the Nm value was estimated using the formula Nm = (1 − *F*_ST_)/(4 *F*_ST_) [57]. To visualize the gene flow, the SankeyNetwork function of the networkD3 R package was employed [54]. This visualization method allows for the representation of flow and connectivity between different groups, providing a clear depiction of gene flow patterns among the 16 pig breeds/populations.

### 4.4. Migration Events and Genetic Introgression Analyses

To investigate the admixture and migration events among the 16 pig breeds/populations in this study, several analyses were conducted using Treemix and *D*-statistics. The details of the methods used are as follows: Treemix analysis was performed using data from 5 Yunnan indigenous pigs and 11 additional pig breeds/populations, with 3 Visayan warty pigs as an out-group [58,59]. We found that a large number of European commercial pig lineages influenced three Saba individuals, and one of the Vietnamese individuals likely belongs to the Diannan small-ear pigs, so when calculating *D*-statistics, we excluded these unavailable individuals for further analysis. For differentiation analysis, the same genotype call set used for estimating *D*-statistics by qpGraph from the AdmixTools package was utilized. *D*-statistics (H1, H2, H3, and Visayan warty pig) were calculated to measure shared drift based on allelic frequency [60,61]. The counts of *D*-statistics computed where Southeast Asian and East Asian pig populations were H3; Visayan warty pig was the out-group, and Diannan small-ear and Diqing Tibetan served as H1 and H2 [61]. In addition, *D*-statistics were estimated with Diannan small-ear as H1, considering all possible combinations of Southeast Asian and East Asian pig populations as H2 and selecting Vietnam, Jeju black, Korean native, Korean wild boar, and Asian wild boar as H3, with Visayan warty pig as the out-group. This analysis helps examine the genetic introgression of Diannan small-ear pigs to other Yunnan pigs. To assess the genetic introgression of Diannan small-ear to other Yunnan pigs, *D*-statistics (H1, H2, Diannan small-ear, and Visayan warty pig) were calculated. Baoshan, Gaoligongshan, Saba, and Diqing Tibetan were considered H1, and all possible combinations of Southeast Asian and East Asian pig populations were tested as H2. *Z*-scores were estimated using a block jackknife approach with 5 Mbp blocks. *Z*-scores help assess the statistical significance of the calculated *D*-statistics, and the events with |Z-score| > 3 were considered to be significant [61]. Finally, qpGraph analysis was conducted on all SNPs from the filtered sites, using the default setting [61].

### 4.5. Genome-Wide Scan for Gene Introgression through fd Statistic

To identify vital signal regions of introgression from Vietnam in the genome of Diannan small-ear pig samples, the following analyses and annotations were performed: (1) Admixture proportions estimation: Admixture proportions between Diannan small-ear pig samples were estimated using the ABBA-BABA-based *fd* statistic in windows of 100 kb [61]. This analysis helps identify regions of the genome that show evidence of introgression from Vietnam to Diannan small-ear pig. (2) Number of SNPs and introgression region plotting: The number of SNPs within 100 kb window size was plotted to visualize the segment of the introgression region by using the CMplot package in R software (v4.2.1). The goal is to highlight the regions where introgression events are occurring. (3) Detection of outlier windows: The top 0.1% of the remaining *fd* values were selected, and outlier windows within 1 Mbp of each other were grouped together into a single signal. This step aims to identify regions with exceptionally high *fd* values, indicating potential introgression [61]. (4) Calculation of genetic statistics: Within the candidate regions, genetic statistics, such as *F*_ST_, PI, and Tajima’s D statistic, were calculated using VCFtools [62].dxy was calculated by popgenWindows.py [61]. These statistics provide insights into genetic differences and diversity within the candidate introgression regions.

### 4.6. Gene Annotation and Enrichment Analysis

We conducted downstream annotation and enrichment analysis of the introgression loci. (1) Annotation of candidate genes: Regions showing high *fd* values and low dxy and *F*_ST_ were considered candidates for multiple-gene introgression. The 20 kb region upstream and downstream of these loci from the pig reference genome (*S. scrofa11.1*) annotation were examined. The pig QTL database from the Animal QTL database was also used to annotate the overlapping introgression regions [63]. (2) Visualization of haplotype distribution: The distribution of haplotypes in different pig populations within the introgression regions and candidate genes was visualized using R software (v4.2.1) [54]. Long extended haplotypes were left in genome due to the selection sweep [6]. To explore whether alleles of introgression genes were subjected to selection in two populations, we used rehh software (v 3.2.2) R package to create a bifurcation diagram, starting from the alleles of introgression genes for two breeds/populations, which were divided according to the value of PC1. (3) LD heatmap for candidate genes: The LD heatmap for candidate genes was generated using the LDBlockShow tool [64,65]. (4) KEGG and GO for candidate genes: To gain a further understanding of the gene functions and signaling pathways of the identified introgression genes from Vietnam pigs to the Diannan small--ear pig population, online KEGG pathway and GO term analyses were conducted using KOBAS 3.0 [66], and the significant threshold was set as a *p*-value < 0.05 (KEGG) and to *p*-value < 0.01 (GO) to detect significantly enriched pathways and terms.

### 4.7. RNA Expression in Human Cell

To analyze the expression of introgressive genes in human cells, we downloaded the cell expression data from The Human Protein Atlas (https://www.proteinatlas.org/, accessed on 16 March 2023) [67,68]. The data were obtained from the human-homology gene, and, based on reliability scores, we predicted the main and additional locations of gene expression in human cells. The RNA expression of subcells in whole organs and tissues was analyzed to determine specific expression patterns. The highest expression levels in organs were used for subsequent single-cell analysis [69]. To visualize different single-cell clusters, cell types, and RNA expression in selected organs, we applied the UMAP (Uniform Manifold Approximation and Projection) method [69]. To further investigate the expression patterns, we plotted a heatmap that shows the expression levels of the introgressed gene and well-known cell-type markers in different single-cell type clusters. The expression levels were measured using nTPM (normalized transcripts per million) and *Z*-score values [69,70]. This allows for a comparison of gene expression levels across different cell types within the tissue. Additionally, we compared the specificity of RNA expression in three cell types: endothelial cells, fibroblasts, and smooth muscle cells. These cell types were used as controls for comparison purposes.

### 4.8. PheWAS, Gene Expression of Bulk Tissue and Mouse Gene Knockout

By integrating data from these various sources, we can gain a comprehensive understanding of the expression, association with complex traits in pigs, potential implications for human traits, and functional characterization of the introgressed genes. To examine the expression level of introgressed genes in different tissues of the pig, we downloaded the gene expression data of 34 tissues from the PigGTEx [27,30]. To further explore whether the introgressed genes were related to the complex traits of pigs, we searched the PheWAS results from the PigBiobank database, which includes 268 complex traits in five main categories (health, reproduction, production, exterior, meat and carcass traits) in pigs [27,30]. Using the human PheWAS of the GWASATLAS database (https://atlas.ctglab.nl/PheWAS/, accessed on 16 May 2023), we searched phenotypes generated by candidate genes in human GWAS results [71], where we kept only the results with a *p*-value < 0.01 and sample size > 5000. To further understand the function of introgressed genes in our study, we phenotypically characterized mice mutants and controls in IMPC data (https://www.mousephenotype.org/, accessed on 10 May 2023) [29,72]. Then, we selected the significant phenotypes to compare the statistical significance between WT and HOM homozygous mice [29,72].

## 5. Conclusions

In summary, this study sheds light on the population structure, differentiation, gene flow, introgression, signature selection, complex traits, and biological function of Yunnan indigenous pigs, highlighting the possible influence of gene flow from Southeast Asian pigs, particularly Vietnam pigs, on the Diannan small-ear pig population. Furthermore, the identification of specific genes associated with important traits, such as fat mass, immunity, and litter weight, contributes to our understanding of the genetic factors underlying these complex traits in pigs. The integration of various databases facilitates a comprehensive analysis of gene function across different species, offering valuable insights into multi-species genetic studies. This approach enabled us to gain insights into the genetic contribution of Southeast Asian pigs to Chinese pigs and provided a novel method for simultaneously analyzing gene function across multiple species in biological databases.

## Data Availability

The newly sequenced 64 whole-genome sequences of Yunnan indigenous pig are available from the National Genomics Data Center with the Bioproject accession numbers PRJCA016012 (https://ngdc.cncb.ac.cn/bioproject/browse/PRJCA016012).

## Supplementary Materials

The following supporting information can be downloaded at: https://www.mdpi.com/article/10.3390/ijms25115689/s1.
