# Supplementary material for: Integrating Multiple Database Resources to Elucidate the Gene Flow in Southeast Asian Pig Populations
Source: Int J Mol Sci. 2024 May 23;25(11):5689. doi: 10.3390/ijms25115689 (PMC11171535; doi:10.3390/ijms25115689)
Supplement: Supplementary file 1 [file ijms-25-05689-s001.zip › Additional file 2-Supplementary figure.pdf]

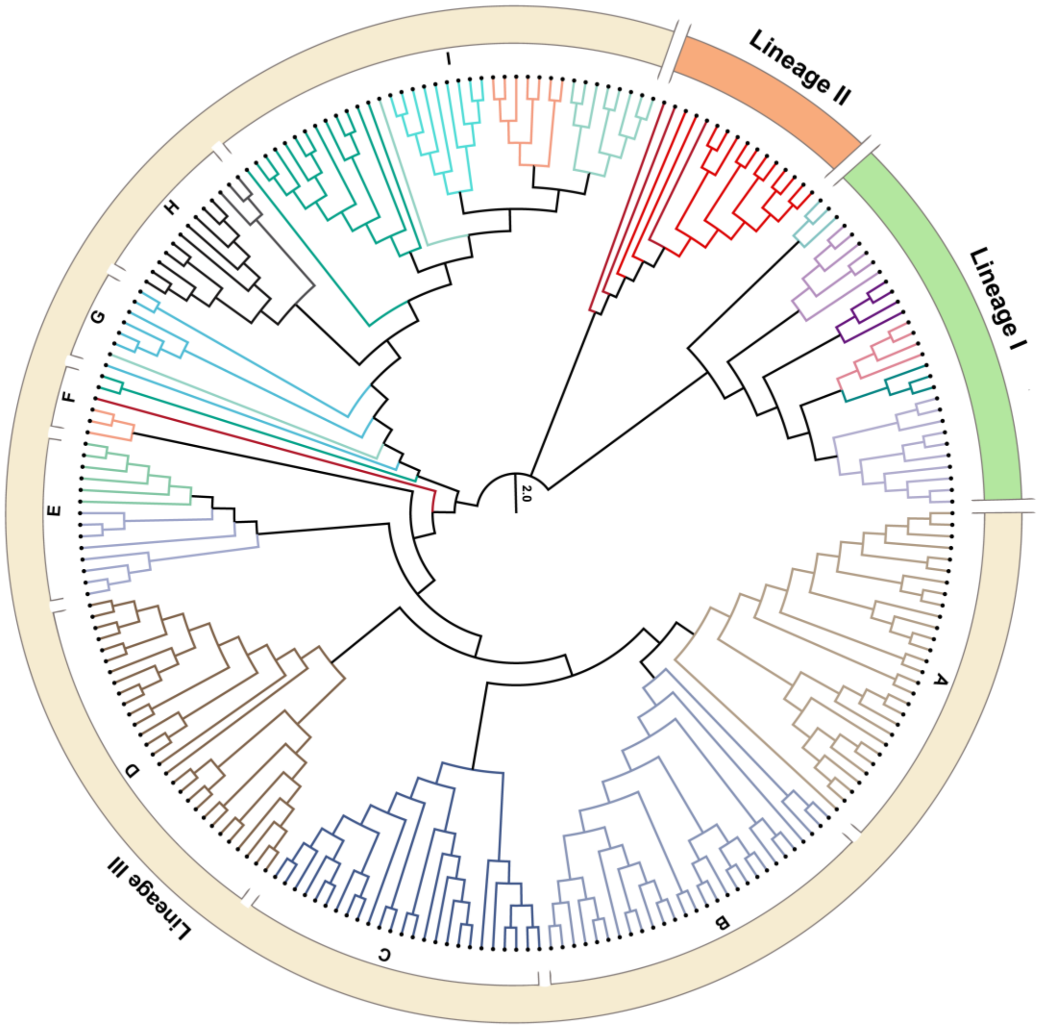

Figure S1. Neighbor-joining (NJ) phylogenetic tree from 236 pigs in three lineages (Lineage I, II, and III) using the whole genome sequencing data. All pigs are plotted with same color (see Figure 1C legend).

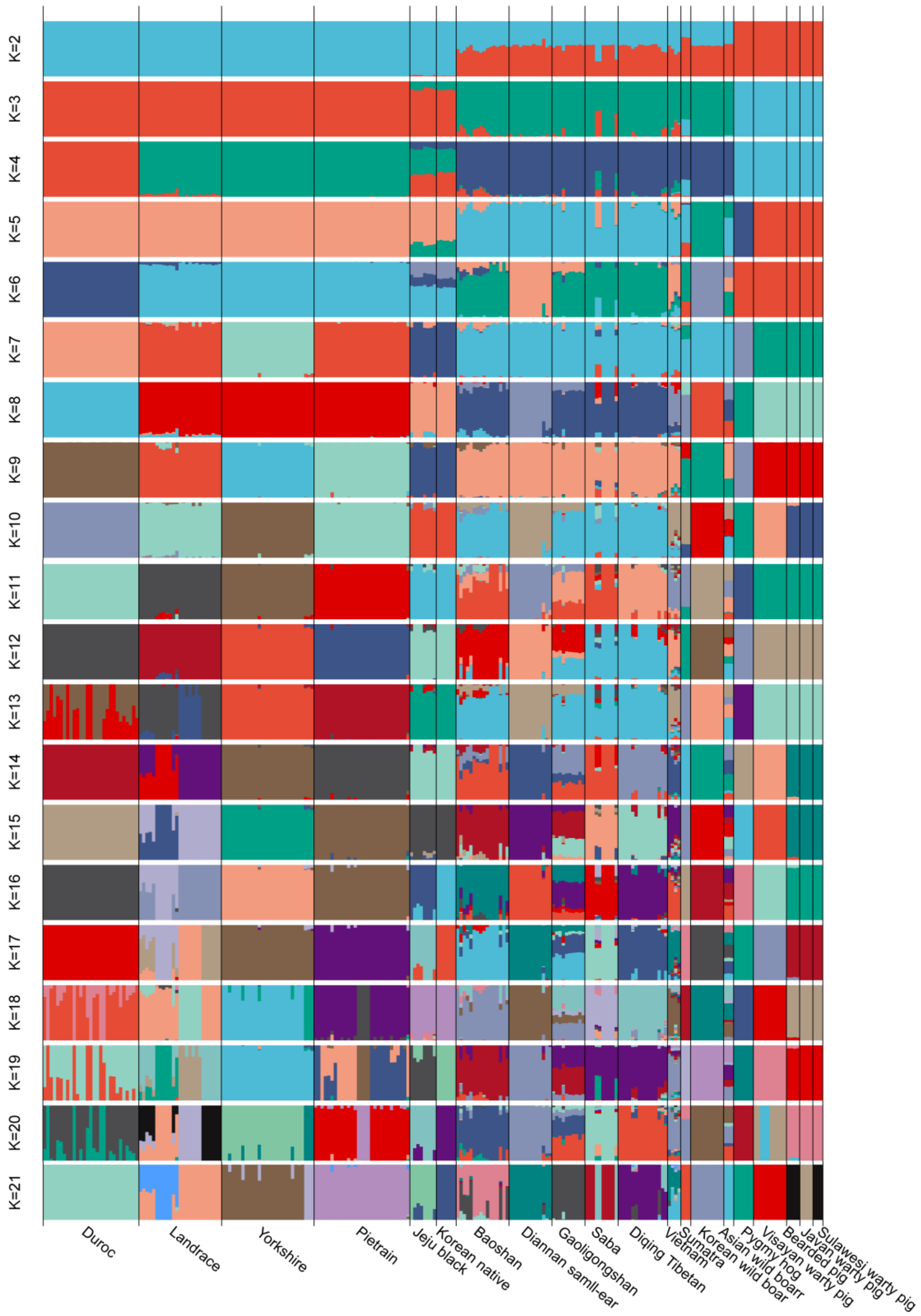

Figure S2. The ancestry compositions of European and Southeast Asian pigs using ADMIXTURE with the assumed number of ancestries from *K*=2 to *K*=21.

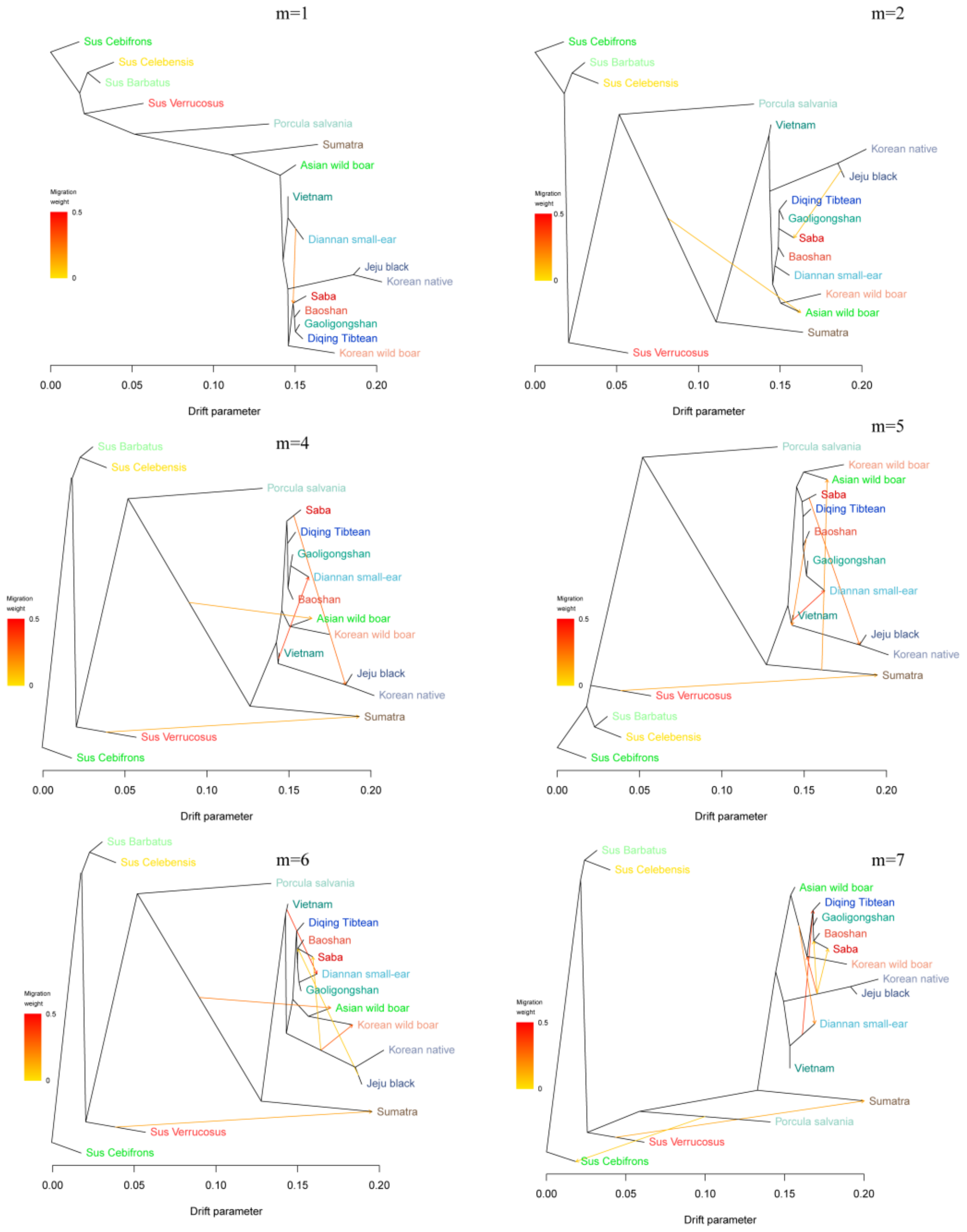

Figure S3. TreeMix analysis revealing gene flow and migration events among 16 pig breeds/populations. Estimated with TreeMix and allowing 1, 2, 4, 5, 6 and 7 migration events. Visayan warty pig (*Sus Cebifrons*) was used as an outgroup to root the trees.

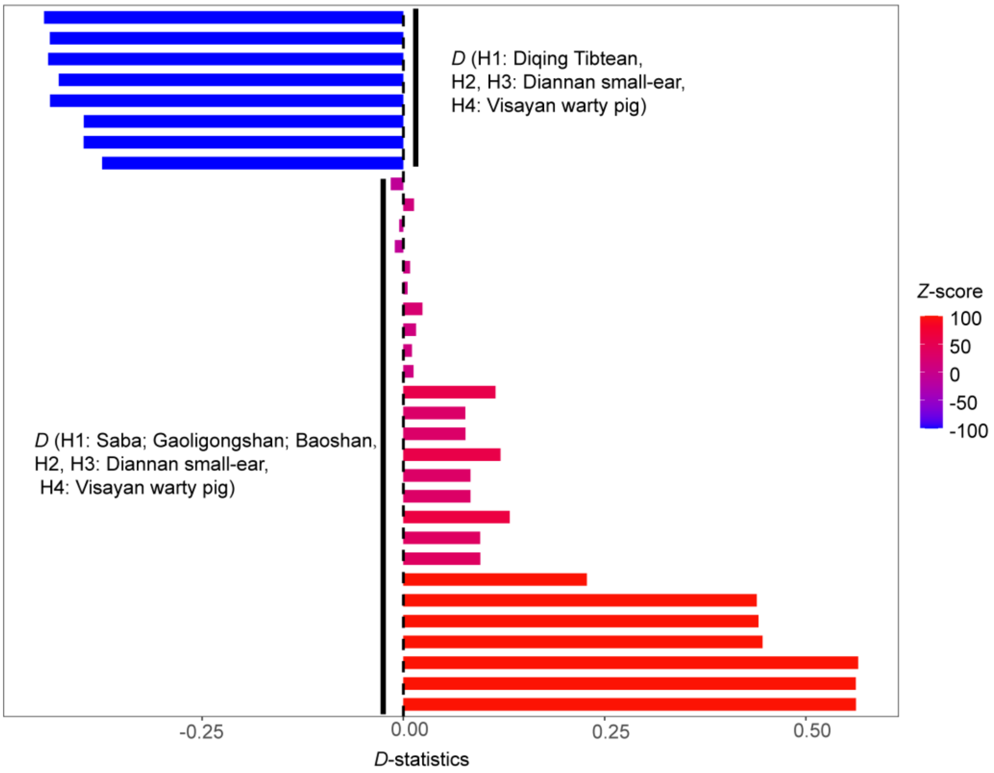

Figure S4. Allele sharing between Diannan small-ear and other Yunnan pigs (Diqing Tibtean, Saba, Gaoligongshan and Baoshan), or Diannan small-ear and H2, H2 represents the Asian wild boar, Diqing Tibetan, Saba, Gaoligongshan, Baoshan, Sumatra, Korean native, Korean wild boar, Vietnam and Jeju black. H4: Visayan warty pig (*Sus Cebifrons*) represents outgroup.

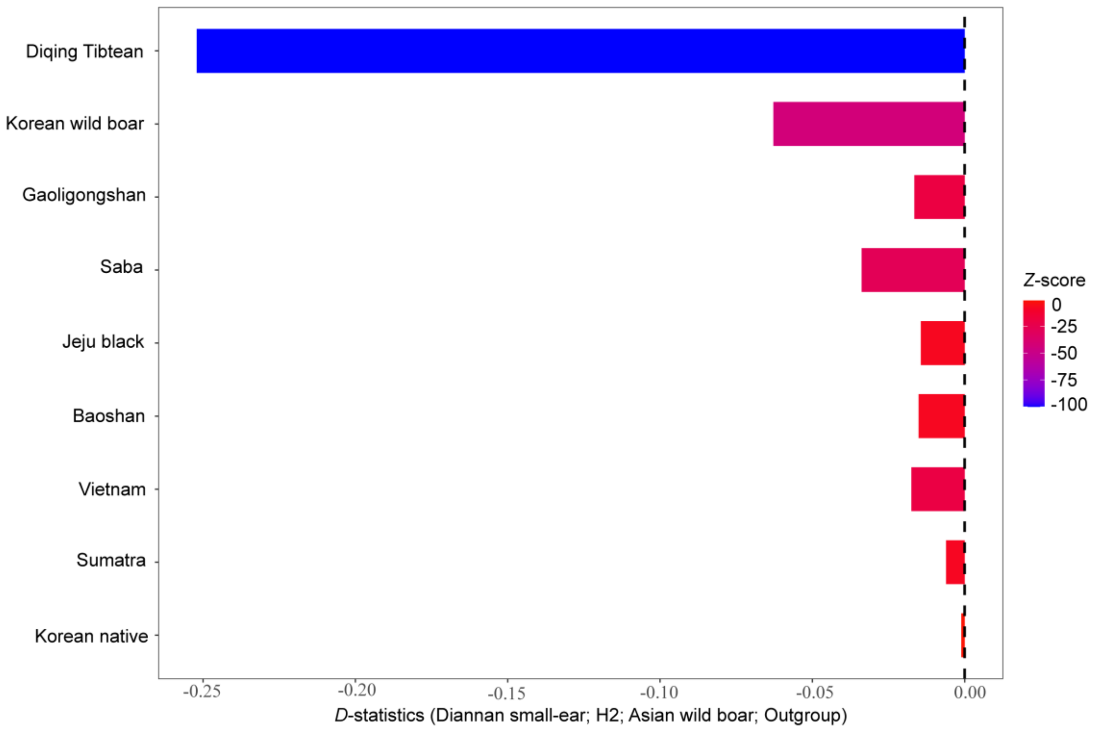

Figure S5. Allele sharing between Asian wild boar and Diannan small-ear, or Asian wild boar and H2, H2 represents the Diqing Tibetan, Saba, Gaoligongshan, Baoshan, Sumatra, Korean native, Korean wild boar, Vietnam and Jeju black. H4: Visayan warty pig (*Sus Cebifrons*) represents outgroup.

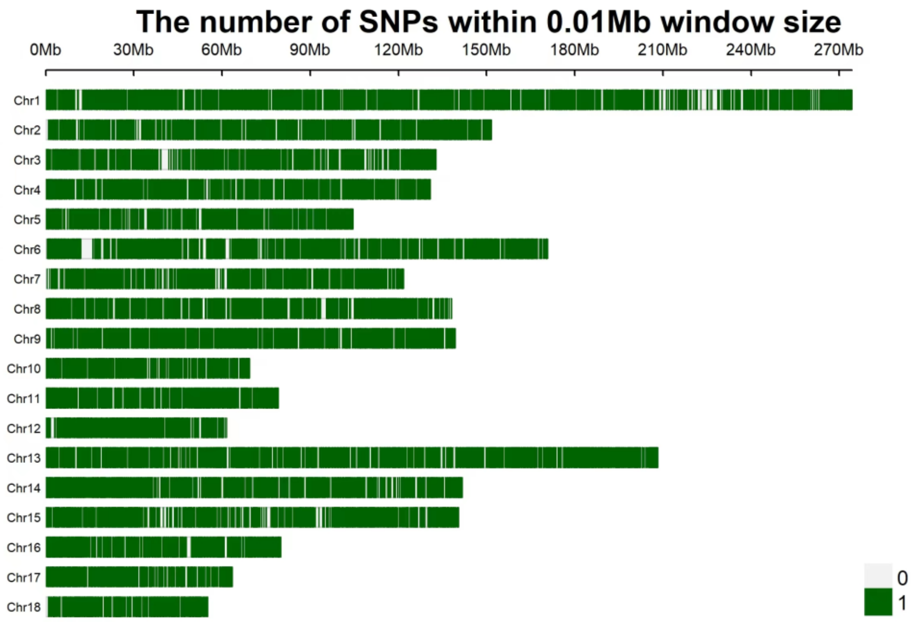

Figure S6. Genome-wide introgression map of Vietnam from Diannan small-ear breed. The number of SNPs within 0.01Mb window size plot of the genome-wide distribution showed the introgression region.

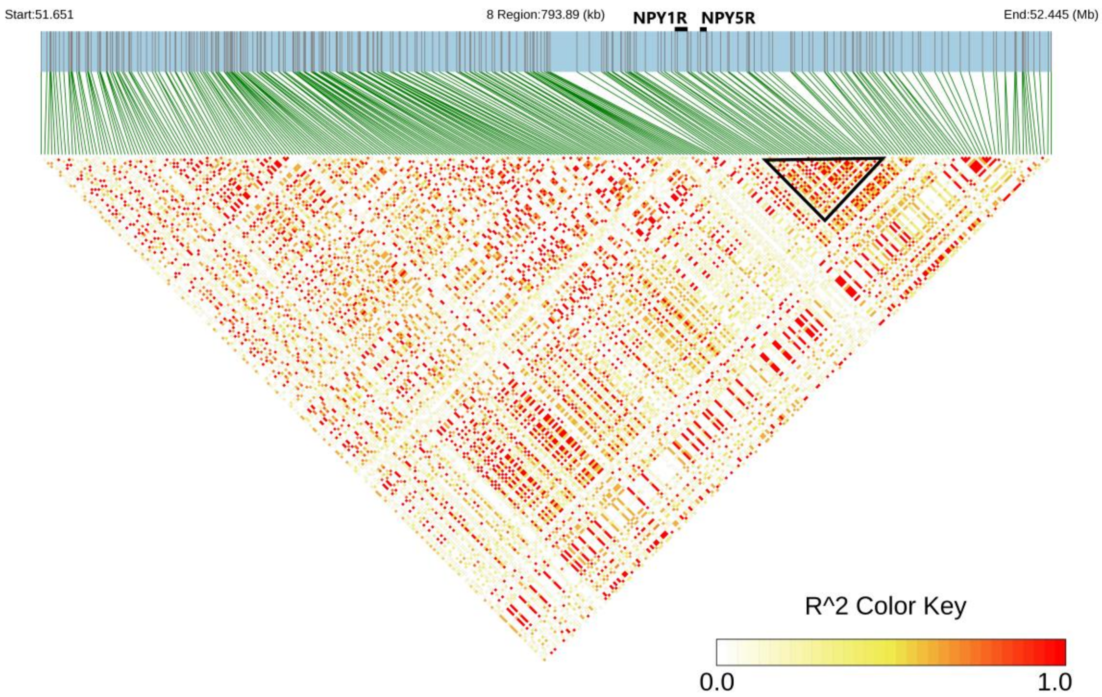

Figure S7. *NPY5R* and *NPY1R* genes in LD block, and a representation of the pairwise R^2 values on chromosome 8 (51.651Mb to 52.445Mb).

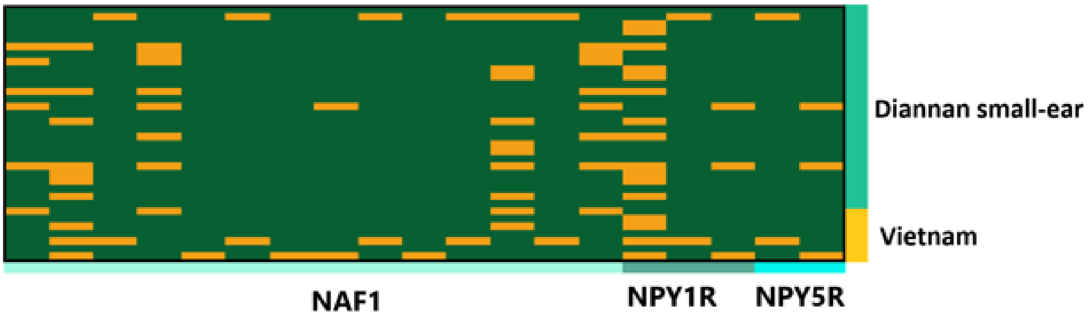

Figure S8. Degree of haplotype sharing between Diannan small-ear and Vietnam pig populationsin three genes (*NAF1*, *NPY1R* and *NPY5R*). The major allele at each SNP position is colored in yellow.

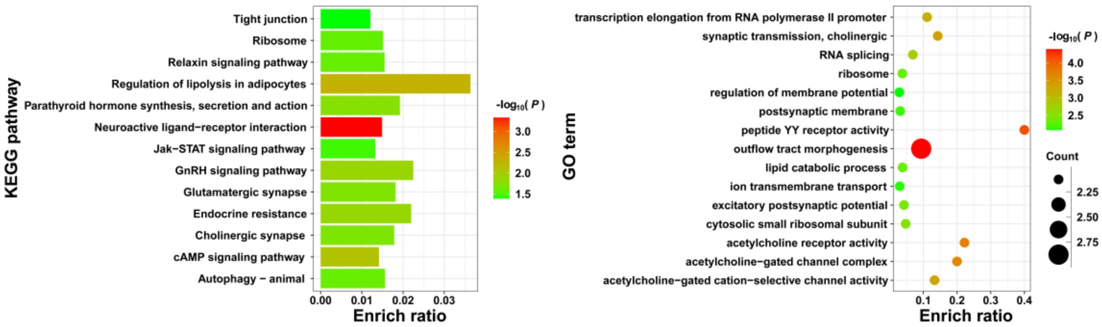

Figure S9. KEGG pathways and GO terms of introgression genes from Vietnam pigs to the Diannan small-ear pig population.

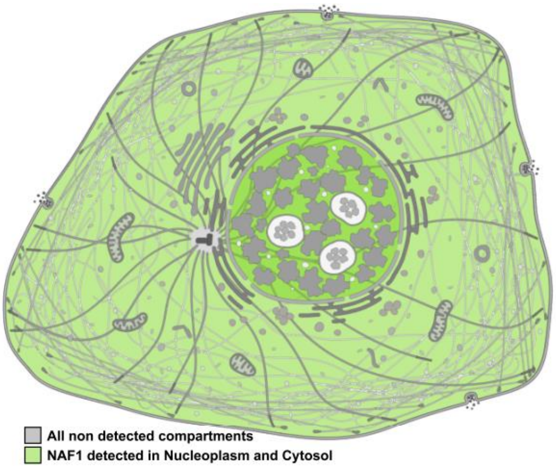

Figure S10. The location (localized to the Nucleoplasm and cytosol) of *NAF1* gene in human cells.

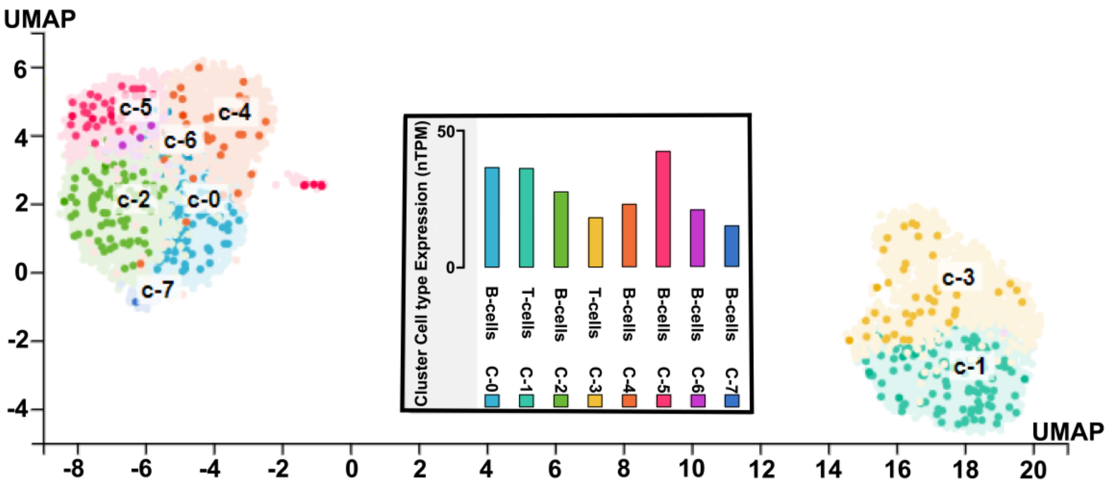

Figure S11. RNA expression in the single cell type clusters identified in lymph nodes tissue visualized by a UMAP plot and a barchart. Colored according to cell type group. Scatter plot, all cells color scale % of max. nTPM: transcripts per kilobase of exon model per million mapped reads.

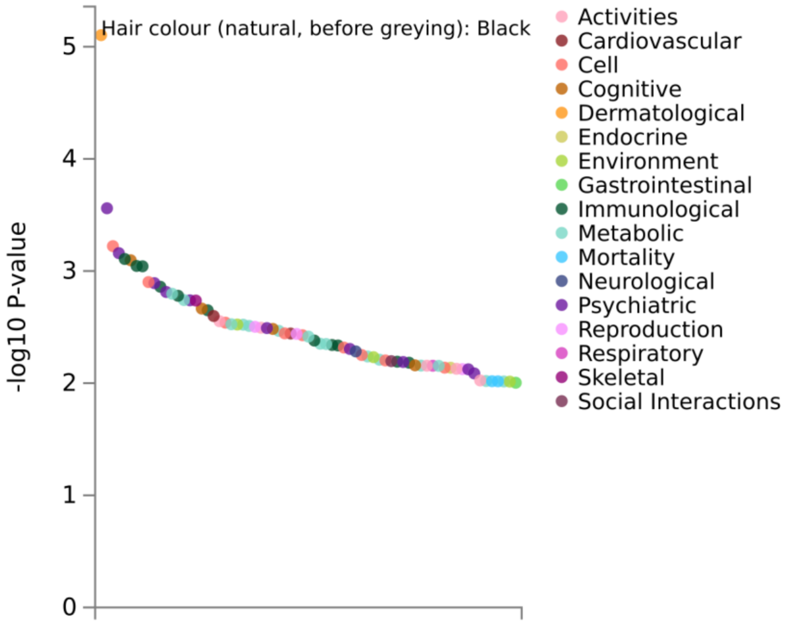

Figure S12. *NAF1* gene in human PheWAS. For SNPs, 0.01 is the maximum *P*-value. Different color is based on phenotype type groups, each consisting of phenotypes with functional features in common.

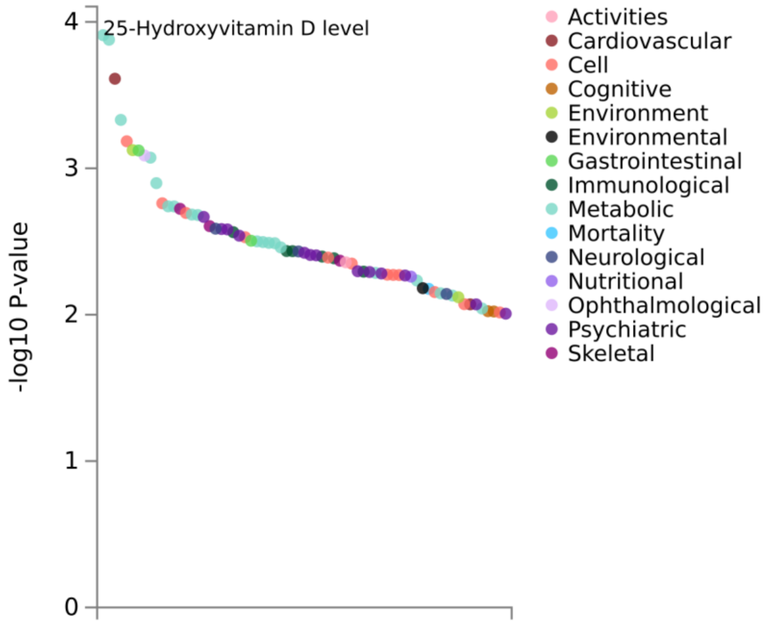

Figure S13. *NPY1R* gene in human PheWAS. For SNPs, 0.01 is the maximum *P*-value. Different color is based on phenotype type groups, each consisting of phenotypes with functional features in common.

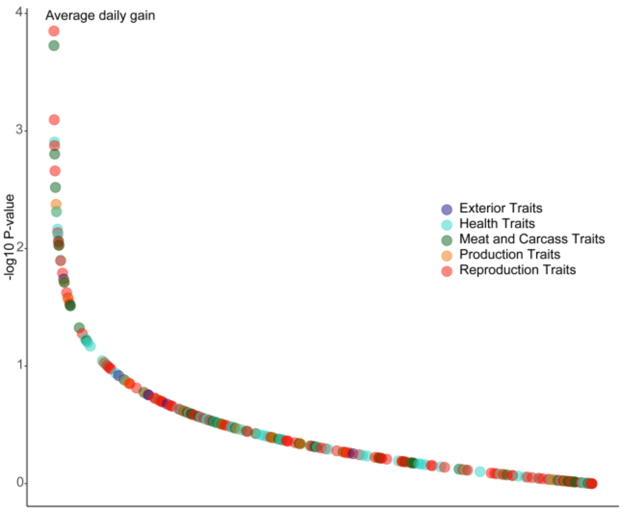

Figure S14. *NAF1* gene in pig GWAS. Different color is based on phenotype type groups, each consisting of phenotypes with functional features in common.

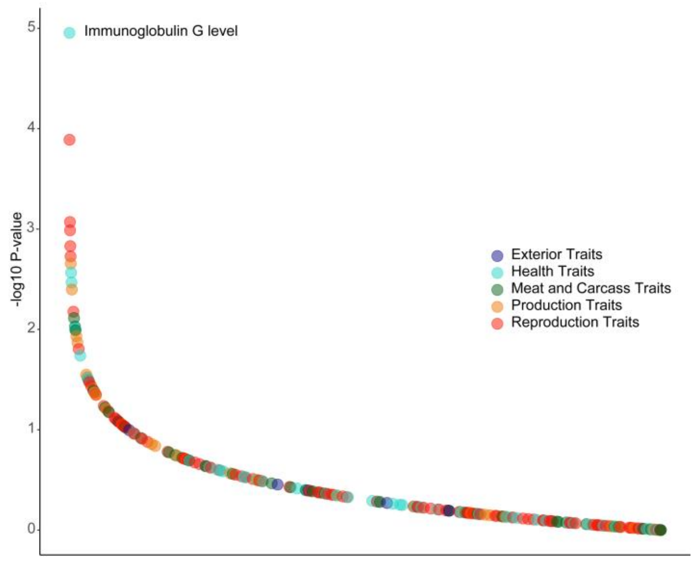

Figure S15. *NPY1R* gene in pig GWAS. Different color is based on phenotype type groups, each consisting of phenotypes with functional features in common.

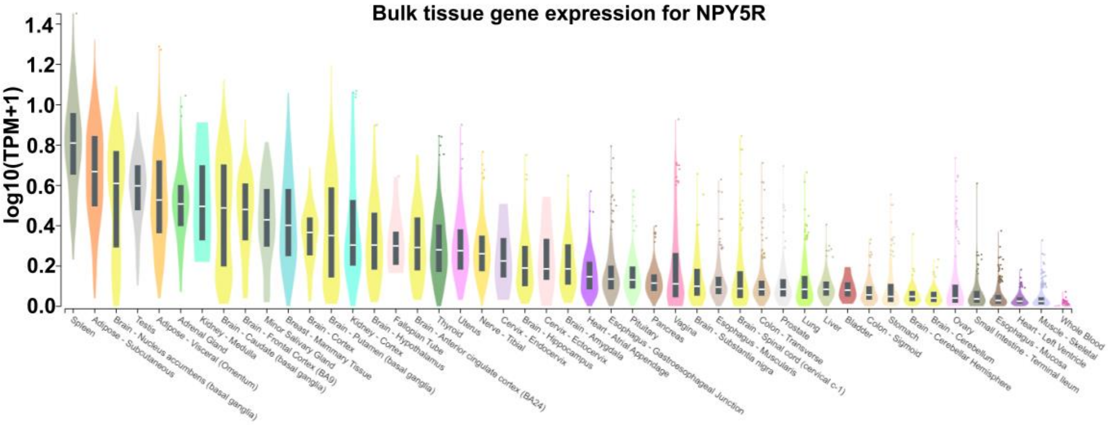

Figure S16. Bulk tissue gene expression for *NPY5R* gene in Human GTEx database.
